# Prognostic factors related to overall survival in adolescent and young adults with medulloblastoma: A systematic review

**DOI:** 10.1093/noajnl/vdac016

**Published:** 2022-02-09

**Authors:** Han Yan, Veda Zabih, Ute Bartels, Sunit Das, Paul Nathan, Sumit Gupta

**Affiliations:** Institute of Health Policy, Management and Evaluation, University of Toronto, Toronto, Ontario, Canada; Division of Neurosurgery, The Hospital for Sick Children, Toronto, Ontario, Canada; Division of Hematology/Oncology, The Hospital for Sick Children, Toronto, Ontario, Canada; Division of Hematology/Oncology, The Hospital for Sick Children, Toronto, Ontario, Canada; Division of Neurosurgery, St. Michael’s Hospital, University of Toronto, Toronto, OntarioCanada; Institute of Health Policy, Management and Evaluation, University of Toronto, Toronto, Ontario, Canada; Division of Hematology/Oncology, The Hospital for Sick Children, Toronto, Ontario, Canada; Institute of Health Policy, Management and Evaluation, University of Toronto, Toronto, Ontario, Canada; Division of Hematology/Oncology, The Hospital for Sick Children, Toronto, Ontario, Canada

**Keywords:** adolescent, medulloblastoma, prognosis, systematic review, young adults

## Abstract

**Background:**

Medulloblastoma is a rare diagnosis among adolescents and young adults (AYA). Though prognostic factors and treatment are well characterized among children with medulloblastoma, equivalent data for AYA are sparse. We conducted a systematic review to identify predictors of survival among AYA with medulloblastoma.

**Methods:**

We searched for primary studies of AYA (age 15–39 at diagnosis) with medulloblastoma in high-income countries within OVID MEDLINE, EMBASE, and EBM Reviews-Cochrane library databases from inception to August 2020. Patient demographics, primary outcomes, and univariate and multivariable data on all prognostic factors were collected from included studies. Prognosticators were characterized as patient, disease, or treatment-related.

**Results:**

We identified 18 articles. 5-year overall survival ranged between 40% and 89%, while disease-free survival ranged from 49% to 89%. Study quality was low as assessed by the Quality in Prognostic factor Studies tool. Though meta-analyses were not possible due heterogeneity, narrative summaries suggested that lower disease burden, superior postoperative functional status, and higher doses and larger fields of radiation were associated with improved survival. Reported chemotherapy regimens were heterogeneous in timing, agents, and relationship with radiation, precluding meaningful comparisons. Only one study included molecular subgroups for analysis, with the majority (76.5%) of tumors classified as Sonic Hedgehog (SHH).

**Conclusions:**

Prognostication and treatment of AYA medulloblastoma is limited by a dearth of primary evidence and lack of specificity for patients aged 15–39. Dedicated prospective trials to delineate the benefit of various chemotherapy and radiation regimens are required in this population to identify prognosticators and ideal treatment regimens.

**Key Points**

- It is unknown whether treatments and prognosticators defined in children with medulloblastoma apply to AYA.
- The existent literature is heterogenous and suffers from methodological flaws.
- Higher doses and larger fields of radiation were associated with improved survival; no conclusions could be made about chemotherapy.

**Importance of the Study**

This narrative systematic review demonstrates that literature on AYA patients with medulloblastoma is sparse, heterogeneous, and is lacking information on molecular subtypes as prognosticators. Among patient and disease related factors, increased extent of disease and poor post-operative functional status were associated with worse prognosis. Among treatment factors, the use of radiation, at larger volume and higher doses, generally favoured improved overall and disease-free survival. The role of chemotherapy remained unclear given the heterogeneity of agents, timing and relationship with radiotherapy. Multi-center collaboration is required to standardize treatment regimens and conduct research specific to AYA with medulloblastoma.

Medulloblastoma in adolescents and young adults (AYA) is rare, representing less than 1% of central nervous system tumors in patients over the age of 16.^1^ As an embryonal tumor of the cerebellum, medulloblastomas are biologically and clinically diverse. Diagnoses of medulloblastoma at different ages suggest differences in tumor biology, and consequently distinct prognostic patterns that should ideally inform treatment guidelines. Despite this, its rarity in AYA means that treatment recommendations are often extrapolated from pediatric cohorts.^2^ The largest retrospective meta-analysis of 907 adult medulloblastoma patients aged >15 years, reported a 5-year overall survival of 50.9%.^3^ An international, prospective study of 270 children younger than age 5 demonstrates a much better 8-year overall survival of 76%.^4^ AYA with medulloblastoma represent a unique cohort distinct from both younger and older patients; factors contributing to differences in survival in this population require dedicated study.

For example, historically, medulloblastoma was categorized histologically (classical, desmoplastic/nodular, large cell/anaplastic), but are now is also classified by four molecular subgroups (wingless-type (WNT), sonic hedgehog (SSH), group 3 and group 4). The prognostic value of molecular subgroups is clear in children although this may not mirror the natural history seen in AYA.^5–7^ Furthermore, there may be differences between AYA and older adults (>40 years) in tumor biology that have never been studied.

In general, AYA with cancer face unique diagnostic and treatment challenges. Oncology care may be less standardized as AYA patients fall between pediatric and adult services, and entry into clinical trials for long-term data may be more difficult.^8,9^ There is currently only one prospective trial on medulloblastoma for adults that has generated longitudinal data.^10^ Moreover, pediatric trials that include patients up to 21 years often have low accrual rates for patients over 14 years.^9,11^ AYA patients require special collaborations between pediatric and adult clinicians and researchers to address their complex needs.

Given the reliance of AYA prognostication and treatment decisions on pediatric evidence, we sought to identify literature specific to medulloblastoma in AYA through a systematic review. The goal of this analysis was to (1) identify the survival rates of AYA with medulloblastoma; and (2) to evaluate the demographic, disease, and treatment-related prognostic factors of survival in AYA (15–39 years at diagnosis) diagnosed with medulloblastoma.

## Methods

### Data Sources and Search Strategy

This review followed the Preferred Reporting Items for Systematic reviews and Meta-Analyses (PRISMA) guidelines.^12^ We searched OVID MEDLINE, EMBASE and EBM Reviews-Cochrane library databases from inception to August 2020. Search terms were developed in consultation with a library scientist. A sample search strategy can be found in Supplementary Appendix 1. 

### Screening and Study Selection

Studies were selected if they met the following inclusion criteria: (1) original research studies that reported predictors of medulloblastoma related to AYA outcomes (eg overall survival, progression-free survival); (2) age at diagnosis was 15–39 years; studies were included if AYA outcomes (15–39) were reported separately, or if AYA patients represented more than 50% of the entire study group; (3) diagnosis of medulloblastoma defined according to WHO 2016 classification; (4) sample size of AYA patients >20 and (5) studies published in English language. We excluded studies conducted in LMICs as defined by the World Bank,^13^ reviews, commentaries, editorials, case series, articles in languages other than English, CNS tumors not listed above, and population-based mortality statistics (ie mortality rate of 2/100,000 per year).

Two authors (V.Z., H.Y.) independently examined titles and abstracts to identify eligible studies. Similarly, two authors (V.Z., H.Y.) reviewed full texts for eligible studies independently and involved a third author if needed to resolve any discrepancies. A kappa coefficient was calculated to estimate level of agreement between reviewers.

### Data Extraction and Analysis

We used the CHARMS-PF checklist (**CH**ecklist for critical **A**ppraisal and data extraction for systematic **R**eviews of prediction **M**odelling **S**tudies-**P**rognostic **F**actors)^14^ to extract data from each article. Study type, sample size, primary outcomes, length of follow up, and detailed statistics on all prognostic factors were collected from each study. The QUIPS (**QU**ality in **P**rognostic factor **S**tudies)^14,15^ tool was used to assess risk of bias.^16–18^ QUIPS assessed six domains of potential bias: study participation, study attrition, prognostic factor measurement, outcome measurement, adjustment for other prognostic factors, and statistical analysis and reporting. For each domain, responses to prompting items are aggregated to judge risk of bias as high, moderate, or low. As study heterogeneity was significant, meta-analyses were not feasible.

## Results

### Search Results

**Figure 1.**
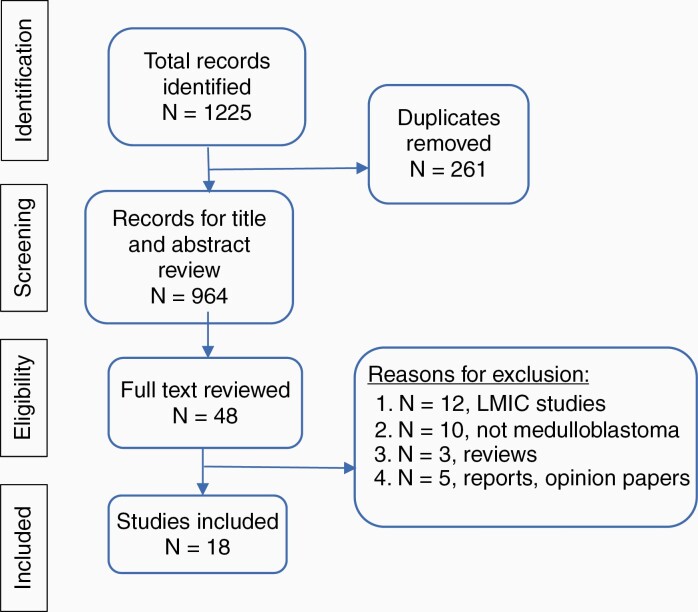
PRISMA flow diagram illustrating study selection process.

Our search identified 1,225 studies. After removing duplicates, the remaining 964 titles and abstracts were screened (Figure 1). Forty-eight studies were identified for full text review. Eighteen studies met inclusion criteria. The kappa measure of agreement between the two reviewers was 90.8% (95% CI 78.4%–100%).

### Study Characteristics and Survival

All 18 studies were published between 1990 and 2018 and were retrospective. Studies were conducted in various countries: 7 in the US, 4 in France, 2 in Germany, 2 in Italy, 1 in Spain, 1 in Canada, and 1 in UK. Sample size ranged from 21 to 751 participants. Included patients ranged in age from 14 to 85 years, though all studies cohorts comprised >50% AYAs. All studies used univariate regression models to identify factors associated with survival; only 8 conducted adjusted/multivariable analyses. Survival outcomes included overall survival, progression-free survival, disease-free survival, event-free survival, and recurrence-free survival. For the purposes of this analysis, we report on overall survival and combined the other outcomes under the term “disease-free survival”. Amongst the included studies, 5-year overall survival ranged between 40% and 89% while disease-free survival was between 49% and 89%. Characteristics of the included studies are shown in Table 1.

**Table 1.** Demographics and Survival Data of Included Studies, *n* = 18

Study. Yr Country	Sample Size	Age at Diagnosis	Length of Follow Up (months)	Overall Survival Rate	Event-Free Survival
Ang C^19^ 2008 Canada	25	Median 30 (18–48)	Median 86 (2–161)	5 y-78% 10 y-30%	5 y-50% 10 y-31%
Aragones M P^20^ 1994 Spain	30	Mean 27 (14-47)	Mean 63	5 y-49% 10 y-36%	5 y-49% 10 y-42%
Atalar B^21^ 2018 US	206	Median 29 (16–66)	Median 31 (0.2–179)	10 y-51%	10 y-38%
Bloom H^36^ 1990 UK	47	Median 24 (16–54)	Minimum 60	5 y-54% 10 y-40%	
Carrie C^22^ 1994 France	156	Median 28 (18–58)	Median 71 (3–211)	5 y-70% 10 y-51%	5 y-61% 10 y-48%
Carrie C^23^ 1993 France	30	Mean 28 (18–44)	Median 104 (range 32–211)	5 y -58.5% 10 y -41%	
Chan A^32^ 2000 US	32	Median 25.5 (16–47)	Minimum 64	5 y-83% 8 y-45%	5 y-57% 8 y-40%
Chargari C^24^ 2010 France	36	Median 27 (16–49)	Median 46 (5–155)	3 y-65.3%	3 y-57.4%
Giordana M^25^ 1995 Italy	44	Median 31 (18–62)	Minimum 36 months	5 y-40% 10 y-35.6%	
Giordana M^35^ 2005 Italy	86	Mean 30.6 (18–72)		5 y-87.9% 10 y-38.6%	
Hadi I^33^ 2018 Germany	21	Median 30.2 (16–45)	Median 92	5 y-89% 10 y-80%	5y-89% 10y-81%
Herrlinger U^26^ 2005 Germany	34	Median 24.5 (16–76)	.	5 y-79% 10 y-56%	
Kann B^27^ 2017 US	751	Median 29 (18–85)	Median 60	5 y-81.6%	
Kunschner L^28^ 2001 US	28	Median 30.5 (19–45)	Median 42 (2–150)	3 y-91% 5 y-84%	3 y-68% 5 y-62%
Lai R^29^ 2008 US	454	Mean 33, 11.1 SD	.	5 y-64% 10 y-50.4%	
Le Q T^30^ 1997 US	34	Median 23(15–56)	Median 111 (34–229)	5 y-58%	
Padovani L^34^ 2007 France	258	Median 29 (18–58)	Median 84	5 y-72% 10 y-55%	5 y-65% 10 y-55%
Rodriguez FJ^31^ 2007 US	74	Mean 32.5 (18–69)	Mean 76 (2–250)	5y-69% 10y-50%	5 y-58% 10 y-50%

### Prognostic Factors

Studies examined numerous variables as possible predictors of outcomes (Supplementary Table 1). Patient-related variables included age,^19–31^ sex,^19–21,24–34^ race,^27,29^ education,^29^ income,^29^ marital status,^29^ insurance,^27^ distance from facility,^27^ year of diagnosis,^29^ Karnofsky Performance Status (KPS) score,^21^ Eastern Cooperative Oncology Group (ECOG) Performance Status,^22,23,33,34^ and the Charlson Deyo Score.^27^ Disease-related variables included histology,^20–23,25–27,29,32,33,35^ tumor location,^20,22,23,25,32,34^ tumor size,^27,29,31^ risk group,^33,34^ extent of disease,^24,29,30^ stage,^19,23,26,27,32^ and involvement of brainstem/4th ventricle floor/CSF.^21–24,26,32,34^ Treatment-related variables included the extent/quality of resection,^19–32,36^ use of chemotherapy,^19,21–24,26,28,31,32,34,36^ and radiation-specific variables.^21,22,25–27,32,33,36^ The most commonly studied prognosticators are reported in Tables 2 and 3.

**Table 2.** Patient and Disease Factors for Overall Survival

	Description	Studies	Comparison	
			Univariate	Multivariate^a^
Patient Factors				
Age		Ang C, 2008,^19^ Aragones M P, 1994,^20^ Bloom H, 1990,^36^ Carrie C, 1993,^23^ Herrlinger U, 2005,^26^ Kunschner L, 2001,^28^ Le Q T, 1997,^30^ Rodriguez FJ, 2007^31^	NS	
		Chargari C, 2010,^24^ Kann B, 2017^27^		NS
	Age ≤ 29 vs. >29	Atalar B, 2018^21^	***P* = .004**	
	Age ≤ 37 vs. >37	Giordana M, 1995^25^	***P* = .05**	
	Age ≥ 20 vs. >20	Lai R, 2008^29^	***P* = .03**	**HR = 2.98, *P* = .035**
Function	KPS ≥ 80 vs. <80	Atalar B, 2018^21^	***P* < .001**	**RR = 2.58, *P* = .01**
	KPS > 90 vs. ≥90	Chargari C, 2010^24^	***P* = .05**	
	Pre-op ECOG ≤ 2 vs. ≥3	Carrie C, 1993^23^	NS	
	Post-op ECOG ≤ 2 vs. PS ≥ 3	Carrie C, 1993^23^	***P* < .0001**	
	ECOG 0-1 vs. 2	Hadi I, 2018^33^	NS	
Disease Factors				
Extent of Disease	Spinal metastases	Atalar B, 2018^21^	NS	
		Lai R, 2008^29^	***P* = .04**	
	Disease beyond posterior fossa vs. not	Chargari C, 2010^24^	***P* = .001**	***P* = .03**
		Le Q T, 1997^30^		**HR = 4.7, *P* = .04**
	4 ventricle floor involvement vs. not	Herrlinger U, 2005^26^	***P* = .03**	
		Padovani L, 2007^34^	***P* = .0002**	**RR = 2.2, *P* = .002**
	Brainstem Involvement vs. not	Padovani L, 2007^34^	***P* = .013**	**RR = 2.7, *P* = .017**
Histology	Classic vs. desmoplastic	Aragones M P, 1994^20^	***P* = .02**	
		Atalar B, 2018,^21^ Bloom H, 1990,^36^ Carrie C, 1993,^23^ Giordana M, 1995,^25^ Herrlinger U, 2005,^26^ Kunschner L, 2001,^28^ Lai R, 2008,^29^ Rodriguez FJ, 2007^31^	NS	
	Classic & desmoplastic vs. unknown	Hadi I, 2018^33^	***P* < .001**	
	Desmoplastic vs. other	Kann B, 2017^27^	***P* = .01**	***P* = .02**
Molecular subgroup	SHH, WNT, Group 4, unknown	Hadi I, 2018^33^	NS	
Chemotherapy Factors				
Use of CT	Yes vs. no	Atalar B, 2018^21^	***P* = .03**	NS
		Carrie C, 1993,^23^ Herrlinger U, 2005,^26^ Padovani L, 2007,^34^ Rodriguez FJ, 2007^31^	NS	
		Chargari C, 2010^24^		NS
	CT + RT vs. RT alone	Bloom H, 1990^36^	***P* < .025**	
		Kann B, 2017^27^	***P* < .001**	***P* = .01**
Radiotherapy Factors				
Radiation	Yes vs. no	Lai R, 2008^29^	***P* = .001**	**HR = .052, *P* = .005**
Posterior fossa RT	< 54 Gy vs. ≥ 54 Gy	Atalar B, 2018^21^	***P* < .001**	
	< 50 Gy vs. ≥ 50 Gy	Padovani L, 2007^34^	***P* < .0001**	**RR = 2.7, *P* = .009**
	55-58 Gy vs. 41-52 Gy	Bloom H, 1990^36^	NS	
Craniospinal RT	No vs. Yes	Atalar B, 2018^21^	***P* < .001**	**RR = 5.32, *P* < .001**
	50 Gy PF + spine vs. <40 Gy on PF	Giordana M, 1995^25^	***P* = .02**	
	≤ 29 Gy vs. > 29	Padovani L, 2007^34^	***P* = .0054**	
	3D-RT vs. 2D-RT	Hadi I, 2018^33^	NS	
	23-30 Gy vs. 30-36 Gy	Kann B, 2017^27^	NS	NS
Time from surgery to RT	≤ 47d vs. > 47d	Atalar B, 2018^21^	***P* = .03**	
	≤ 73d vs. > 73d	Hadi I, 2018^33^	***P* = .031**	
	<5m vs. ≥ 5m	Herrlinger U, 2005^26^	NS	
	Time, days	Kann B, 2017^27^	NS	NS
Duration of RT	≤ 45d vs. > 45d	Padovani L, 2007^34^	NS	
RT boost	Yes vs. no	Atalar B, 2018^21^	***P* < .001**	
Surgery Factors				
Extent of resection		Ang C, 2008,^19^ Aragones M P, 1994,^20^ Kann B, 2017,^27^ Giordana M, 1995,^25^ Herrlinger U, 2005,^26^ Kunschner L, 2001,^28^ Le Q T, 1997,^30^ Rodriguez FJ, 2007^31^	NS	
	Complete vs. not	Atalar B, 2018^21^	***P* = .01**	
		Carrie C, 1993^23^	***P* < .05**	
		Bloom H, 1990^36^	***P* < .005**	
		Chargari C, 2010^24^	***P* = .04**	
	Gross total vs. biopsy	Lai R, 2008^29^	***P* = .012**	**HR = 0.33, *P* = .012**

Abbreviations: CT, computed tomography; ECOG, Eastern Cooperative Oncology Group; KPS, Karnofsky Performance Status; NS, not significant; PF, posterior fossa; PS, performance status; RT, radiation therapy; SSH, sonic hedgehog; WNT, wingless.

^a^Multivariate ratios represent risk for shorter overall survival of the specified factor.

**Table 3.** Patient-related Factors for Disease-free Survival

	Description	Studies	Comparison	
			Univariate	Multivariate^a^
Patient Factors				
Age		Carrie C, 1994,^22^ Kunschner L, 2001^28^	NS	
	Age <25 vs. ≥35	Herrlinger U, 2005^26^	***P* = .005**	
	Age (continuous)	Le Q T, 1997^30^		**HR = 0.91, *P* = .01**
Function	Pre-op ECOG ≤2 vs. PS >3	Carrie C, 1994^22^	***P* = .03**	NS
	Post-op ECOG ≤2 vs. PS ≥3	Carrie C, 1994^22^	***P* = .0001**	**RR = 4.3, *P* = .003**
		Padovani L, 2007^34^	***P* < .0004**	
	ECOG 0-1 vs. 2	Hadi I, 2018^33^	NS	
Disease Factors				
Extent of Disease	4th ventricle floor involvement vs. not	Carrie C, 1994^22^	***P* = .0004**	**RR = 2.5, *P* = .005**
		Padovani L, 2007^34^	***P* = .0002**	
		Herrlinger U, 2005^26^	NS	
	Disease beyond posterior fossa vs. not	Chargari C, 2010^24^	***P* = .02**	
		Le Q T, 1997^30^		**HR = 7.8, *P* = .005**
	Metastases vs. none	Padovani L, 2007^34^	***P* = .046**	
Histology	Classic vs. desmoplastic	Carrie C, 1994^22^	***P* = .03**	**RR = 2.9, *P* = .02**
		Chan A, 2000,^32^ Herrlinger U, 2005,^26^ Kunschner L, 2001^28^	NS	
	Classic & desmoplastic vs. unknown	Hadi I, 2018^33^	***P* < .001**	
Molecular subgroups	SHH, WNT, Group 4, unknown	Hadi I, 2018^33^	NS	
Chemotherapy Factors				
Use of CT	Yes vs. no	Carrie C, 1994,^22^ Chan A, 2000,^32^ Herrlinger U, 2005^26^	NS	
Radiotherapy Factors				
Craniospinal RT	<30 Gy total vs. ≥30 Gy	Carrie C, 1994^22^	***P* = .003**	**RR = 4.3, *P* = .05**
	3D-RT vs. 2D-RT	Hadi I, 2018^33^	NS	
Posterior fossa RT	≤73d vs. >73d	Hadi I, 2018^33^	***P* = .031**	NS
	<5m vs. ≥5m	Herrlinger U, 2005^26^	NS	
Whole Brain	<30 Gy total vs. ≥30 Gy	Carrie C, 1994^22^	NS	
Time from surgery to RT	<5m vs. ≥5m	Herrlinger U, 2005^26^	NS	
Duration of RT	<48d vs. ≥48d	Chan A, 2000^32^	NS	
	≤73d vs. >73d	Hadi I, 2018^33^	***P* = .049**	
Surgery Factors				
Extent of resection	Complete vs. not	Carrie C, 1994,^22^ Herrlinger U, 2005,^26^ Kunschner L, 2001,^28^ Le Q T, 1997,^30^ Padovani L, 2007^34^	NS	
		Chan A, 2000^32^	***P* = .01**	***P* = .02**
		Chargari C, 2010^24^	***P* = .004**	

Abbreviations: ECOG, Eastern Cooperative Oncology Group; PS, performance status; NS, not significant; RT, radiation therapy; SSH, sonic hedgehog; WNT, wingless.

^a^Multivariate ratios represent risk for shorter disease-free survival of the specified factor.

#### Patient-related factors.

—In adjusted multivariate models, factors that were significantly associated with *superior* survival included younger age at diagnosis,^29^ female sex,^30^ private insurance,^27^ diagnosis after 1980,^29^ or superior measure of performance status.^21–24^ Three studies^21,25,29^ reported younger age as a significant predictor of overall survival in univariate analyses, although 10 studies found that age did not have an effect.^19,20,23,24,26–28,30,31,36^ Patient functional status was reported with the Karnofsky Performance Status (KPS) or Eastern Cooperate Oncology Group (ECOG) performance scale. Four studies^21–24^ correlated better function with better overall survival or disease-free survival; specifically postoperative ECOG ≤ 2 (ambulating >50% of the time) was shown repeatedly to be a highly significant predictor of superior outcome.

#### *Disease-related* f*actors.*

—Factors that were significantly associated with *inferior* prognosis in adjusted models were desmoplastic histology (vs classic/other histology),^27^ large cell histology (vs classic medulloblastoma),^29^ extent of disease beyond posterior fossa,^24^ 4th ventricle involvement,^22,34^ and brainstem involvement.^34^ Limited extent of disease at presentation^30^ was associated with better survival.

Comparisons across studies were possible for outcomes grouped under extent of disease and histology (Tables 2 and 3). Extent of disease was categorized with different anatomical boundaries: spine, posterior fossa, 4th ventricle floor, and brainstem. Extent of disease was a significant predictor of inferior prognosis in five^24,26,29,30,34^ of the six studies reporting on overall survival and four^22,24,30,34^ of the five studies reporting on disease-free survival. The most common comparison of histology was between desmoplastic and classic histologies, with the majority of studies reporting a nonsignificant association on univariate analysis for both overall and disease-free survival. Only Hadi et al.^33^ reported on molecular subgroups; 76.5% of tumors were classified as SHH, and no significant survival difference between molecular subgroups (WNT, group 4) was identifiable.

#### Treatment-related factors.

—Complete resection^32^ and gross total resection^29^ were significantly associated with better survival in adjusted models. In studies that reported analyses, amongst the 13 studies reporting on extent of surgical resection and overall survival, eight found no association.^19,20,25–28,30,31^ Similarly, four of the six^22,26,28,30,34^ studies that evaluated the impact of complete surgical resection on disease-free survival showed no relationship on univariate analysis.

Chemotherapy regimens were heterogenous with respect to timing (ie, concomitant, before, or after radiotherapy), duration and which agents were administered. Multivariable analyses of the role of chemotherapy were extremely limited; only one study reported improved overall survival with use of chemotherapy and radiotherapy compared to radiotherapy alone after adjusting for other covariates.^27^ Two other studies reported nonsignificant results regarding chemotherapy use in multivariate analyses.^21,24^ Among studies that reported univariate analyses, two studies^21,36^ found that chemotherapy was significantly associated with survival whereas four other studies did not.^23,26,31,34^ All three studies that compared chemotherapy and disease-free survival showed no significant association.^22,26,32^

Multivariable analyses of the role of radiation were also limited. Craniospinal irradiation,^21^ spinal axis radiation dose > 30Gy,^22^ and radiation versus no radiation^29^ were associated with better survival after adjusting for other covariates. Dose of radiation <50 Gy limited to the posterior fossa was associated with inferior survival.^34^ The role of radiation was studied in univariate analyses in multiple studies. One registry study compared patients that received radiation to those that did not,^29^ while another examined the role of boost doses to the posterior fossa and standard craniospinal irradiation (CSI).^21^ Several studies analyzed different doses of radiation to the posterior fossa or the entire cranial–spinal axis.^21,25,27,33,34,36^ The majority of these concluded that using radiation, radiation boosts, and higher doses were associated with better overall and disease-free survival. Two studies^21,33^ supported an association between shorter time between surgery and radiation and improve overall survival while two studies^26,27^ found no association. Herrlinger et al.^26^ found no association with a threshold of <5 or ≥5 months, and Kann et al.^27^ defined time to RT as a continuous variable. Padovani et al.^34^ demonstrated no difference between having duration of radiation therapy ≤45 days compared to >45 days for overall survival.

### Quality Assessment

Most of the studies did not report any methodological limitations, making it challenging to assess risk of bias. Relying instead on the QUIPS tool, we found that the majority of included studies (15/18) contained one domain at high risk of bias. Only 8 studies conducted multivariable analyses that adjusted for confounders/covariates. Other key methodological limitations included small sample size, selective reporting (reporting only significant factors), and incomplete reporting (magnitude of associations not reported in all studies). Additionally, several studies examined cohorts assembled over an extended period of time,^23,36^ resulting for example in cohorts treated prior to adoption of magnetic resonance imaging. Although we included only studies with >50% AYA, the age range for most studies included older populations and did not report on AYA -specific subgroup analyses. Table 4 provides details on the risk of bias assessment.

**Table 4.** Results of Risk of Bias Assessment Using QUIPS Tool

	1. Study Participation	2. Study Attrition	3. Prognostic Factor Measurement	4. Outcome Measurement	5. Adjustment for Other Prognostic Factors	6. Statistical Analysis and Reporting
Ang C^19^	Moderate	Moderate	Moderate	Low	Moderate	High
Aragones M P^20^	High	Moderate	High	Moderate	Moderate	High
Atalar B^21^	High	Moderate	High	Moderate	Moderate	High
Bloom H^36^	Moderate	Moderate	Low	Low	Moderate	High
Carrie C^22^	High	Moderate	Moderate	Moderate	Moderate	Moderate
Carrie C^23^	Moderate	High	Moderate	Low	High	High
Chan A^32^	Moderate	Moderate	Moderate	Low	Moderate	Moderate
Chargari C^24^	Moderate	Moderate	High	Low	Moderate	Moderate
Giordana M^25^	High	High	Moderate	Low	Moderate	Moderate
Giordana M^35^	High	High	Moderate	Moderate	High	High
Hadi I^33^	High	Moderate	Moderate	Moderate	Moderate	High
Herrlinger U^26^	Moderate	Moderate	Moderate	Low	Moderate	High
Kann B^27^	High	Moderate	Low	Moderate	Low	Moderate
Kunschner L^28^	High	High	Moderate	Low	Moderate	High
Lai R^29^	Moderate	Moderate	Low	Low	Moderate	Moderate
Le Q T^30^	High	Moderate	Moderate	Low	Moderate	Moderate
Padovani L^34^	Moderate	Moderate	Moderate	Low	Low	Moderate
Rodriguez FJ^31^	High	Moderate	Low	Low	High	High

High/Moderate/Low: Indicates High/Moderate/Low risk of bias.

## Discussion

This systematic review identified 18 articles that reported on predictors of overall survival and disease-free survival in AYA patients with medulloblastoma in high-income countries. Regrettably, data are sparse for this specific population. Nonetheless, study results suggested that increased extent of disease^24,26,29,30,34^ and poor postoperative functional status^21–24^ were associated with poorer overall and disease-free survival. Among treatment factors, the use of radiation, at larger volume and higher doses, generally favored improved overall and disease-free survival.^21,22,25,27,29,33,34,36^ Complete resection was not always a clear predictor of better response although the definition of residual disease was inconsistent between studies and lacked granularity.^19,20,25–28,30,31^ The role of chemotherapy remained unclear given the heterogeneity of agents, timing, and relationship with radiotherapy.

### Prognostication

Prognostication of medulloblastoma in adults and children have notable differences. In children, risk stratification is often based on the extent of disease, age greater or less than 3 years, and histopathologic or molecular classifications. Infants, or children younger than 3 years, tend to have poorer survival than older children due to omitted or reduced radiotherapy.^37^ The 5-year survival in children ages 0–5 ranges from 30% to 60%.^4,38,39^ Notably, infants with desmoplastic histology, compared to classic or anaplastic, have demonstrated excellent survival rates up to 90% with chemotherapy alone.^38,40,41^ The prognostic value of histopathology for adults is not as well established. Molecular classification also impacts the prognosis of infants, children and adults differently. Adult medulloblastoma may only comprise three of the four molecular groups, with group 3 tumors being extremely rare.^6,42^ The WNT subgroup occurs in children >4 years and adults, although only children in this subgroup show favorable outcomes, an association not always seen in patients older than 16.^5,6^ Most adult medulloblastoma have classic or desmoplastic histology; 50–60% belong to the SSH group, often involving mutations in *PTCH1* or *SMO*.^42,43^ Furthermore, SHH tumors with a *TP53* mutation are distinctly found in children age 5–18 and impart a poor overall survival.^44^

Identifying prognosticators among AYA with medulloblastoma may require different considerations than in children.^45^ Our review did not identify a specific age within the AYA age range that could be useful for risk stratification. The delineation between infants and children is largely driven by the urgency to reduce total radiation dose in the developing nervous system at a younger age. This is less of a concern in adults, though an age-maturation perspective must be considered for AYA since the brain continues to develop connections into the mid-20s and irradiation can cause endocrinopathies, vasculopathies, and second malignancies. Furthermore, the higher mutation rate in SHH subgroup in adults raises the question of whether an age-mutation rate relationship exists.^46,47^ On the other hand, extent of disease, measured by either primary tumor or metastases specific staging, did seem to predict prognosis among AYA.^24,26,29,30,34^ In contrast to pediatric cohorts, the majority of studies did not find that AYA with desmoplastic histology fared differently than those with classic histology. However, “classic histology” may itself represent a different group among children vs AYA, with the latter more often belonging to the SHH group.^42,43^ Most importantly, there was a clear dearth of literature regarding the impact of molecular groups among AYA population. Prognosis based on molecular subtypes evidently differ between children and adults with medulloblastoma,^6^ and only one study including molecular subgroups was under-powered to find a difference.^33^

### Treatment

In average and high-risk children, multimodal therapy is considered standard, including surgery, adjuvant radiation, and chemotherapy. Average-risk children initiate radiotherapy 4–6 weeks after surgery with 54 Gy to the posterior fossa or local tumor bed and 23.4 Gy CSI.^48,49^ High-risk children receive 36 Gy to the CSI. Chemotherapy includes agents such as cisplatin, lomustine, cyclophosphamide, etoposide, and vincristine.^50^ Current adult recommendations are guided mostly by pediatric trials. Average-risk adults with no metastases, small residuals, and classic or desmoplastic histology are recommended by the National Comprehensive Cancer Network (NCCN) to receive 30–36 Gy CSI with a boost to the tumor site of 54–55.8 Gy, with or without chemotherapy.^2^ High-risk adults with residual tumor, disseminated disease, or large cell/anaplastic histology are recommended to have CSI with concurrent chemotherapy and additional postradiation chemotherapy.^2^ The typical regimen following weekly vincristine during CSI includes cisplatin, cyclophosphamide, and vincristine.^2,11^

Our systematic review provided limited and inconsistent evidence supporting these guidelines. Evidence for the prognostic value of extent of surgical resection was, for example, mixed. The best evidence in children accounted for molecular subgroups and measured residual disease <1.5 cm^2,51^ whereas the studies in our analysis provided coarse or subjective classifications of complete/incomplete or subtotal/total. An analysis of 787 patients demonstrated that only patients with group 4 tumors showed a survival benefit with gross total resection, whereas patients with WNT, SHH, or group 3 had no significant survival benefit.^51^ However, safe surgical resection still remains the standard of care and stronger evidence is required before surgical management guidelines can be altered. Second, the use of chemotherapy in AYA has not been clearly shown to work best alongside, or in addition to radiotherapy according to our included studies. Although the beneficial role of chemotherapy in pediatrics has been demonstrated both for survival and radiation reduction, we could not draw meaningful conclusions for the AYA population in this review. Research determining whether pediatric chemotherapy protocols lead to better survival and functional outcomes for AYA with medulloblastoma, or whether regimens using alternative agents or timing may be superior, is needed. Evidence was strongest for the role of higher radiation doses and larger fields. Similar to the NCCN recommendations,^2^ Padovani et al.^34^ observed a benefit of ≥50 Gy to the posterior fossa. The benefit of CSI on overall survival and disease-free survival was seen in two adjusted analyses,^21,22^ again supporting the current recommendations of the NCCN.^2^ However, whether higher dose and larger fields of radiation are required in the context of appropriate chemotherapy containing regimens is still unknown. Evidence in children has demonstrated similar survival outcomes between tumor bed radiation boost instead of posterior fossa boost when combined with chemotherapy. Given ongoing brain maturation in AYA patients and a substantial risks of late effects, this must be a key area for future study.

### Limitations

Our systematic review was limited by the small number of studies in high-income countries with AYA-specific data and their heterogeneity which prevented any formal meta-analyses. The quality of studies was also a concern, with the majority found to be at high risk of bias in at least one domain. For example, most studies were not of sufficient size to conduct multivariable analyses. Studying individual patient and treatment factors as independent predictors of outcomes may lead to misleading results given the complexity of medulloblastoma treatment. The broad range of publication dates, as early as 1990, was necessary given the paucity of literature in this field, but further contributes to heterogeneity and limits the generalizability to current practice. Furthermore, many studies were published prior to the inclusion of molecular subgroups, precluding adjustment on an additional important predictor. Finally, though we included only studies with at least 50% AYA participants, outcomes were generally still combined with those of older patients. Therefore, results may still to some degree reflect adult instead of AYA medulloblastoma.

Future research must compile larger cohorts of AYA with medulloblastoma in order to conduct analyses that account for all important potential prognosticators, including molecular subgroups. Although other prognostic factors may be more important than an arbitrary assignment of an age range, AYA patients with medulloblastoma are understudied compared to children and younger adolescents. Hence, dedicated studies are required in order to prove this hypothesis. In addition, survival endpoints may be insufficient in AYA. Functional outcomes and quality of life measures associated with different treatment strategies should also be studied. There are already dedicated studies and platforms which aim to understand the unique molecular gap between pediatric and adult oncology; this will be crucial in order to improve the outcomes for AYA medulloblastoma research.^52^ In recently published proposed additions to the NCCN adult medulloblastoma guidelines,^53^ the promotion of participation in clinical trials and registries and referral to specialized centers was encouraged as a key step towards this goal.

## Conclusions

The literature on AYA patients with medulloblastoma is sparse, heterogeneous, and does not incorporate information on molecular subtypes as possible predictors of outcome. Though several studies identified that higher doses and larger fields of radiation as associated with improved cancer outcomes, the effects of radiation on cognition and quality of life were not studied over time. The role of chemotherapy and extent of resection require granularity in reporting and standardization in methodologies to understand their impact in the AYA population. Analyses were severely limited by methodological limitations, particularly the inability to adjust for multiple potential prognosticators. Larger and more modern cohorts, ideally in the form of prospective trials, are required in order to improve AYA medulloblastoma outcomes.

## Supplementary Material

vdac016_suppl_Supplementary_Table_S1

vdac016_suppl_Supplementary_Appendix
