# Supplementary material for: Prognostic factors related to overall survival in adolescent and young adults with medulloblastoma: A systematic review
Source: Neurooncol Adv. 2022 Feb 9;4(1):vdac016. doi: 10.1093/noajnl/vdac016 (PMC9161716; doi:10.1093/noajnl/vdac016)
Supplement: vdac016_suppl_Supplementary_Table_S1 [file vdac016_suppl_supplementary_table_s1.docx]

**Supplementary Table 1: studies reporting treatment-related factors associated with survival**

| **Study. Yr**  **Country. Design** | **Baseline Chemotherapy and radiation information** | **Chemotherapy** | | **Radiotherapy** | | **Surgery (tumor resection)** | |
| --- | --- | --- | --- | --- | --- | --- | --- |
|  |  | **Univariate** | **Multivariate** | **Univariate** | **Multivariate** | **Univariate** | **Multivariate** |
| Ang C^19^  2008  Canada  Retrospective | Rad (50–56 Gy): post fossa=19  Rad (23.4–28.8 Gy) craniospinal axis=3  Rad (35–39.6 Gy) craniospinal axis=16  Concurrent chemo-rad =7  Adjuvant chemo=13 | Chemotherapy-nonsig  time between surgery and adjuvant chemo-nonsig | . | . | . | extent of resection-nonsig | . |
| Aragones M P^20^  1994  Spain  Retrospective | Whole brain rad: 28  Rad: post fossa (55Gy)=28  Rad: craniospinal axis=26  Adjuvant Chemo: 8 | . | . | . | . | extent of surgical removal-nonsig | . |
| Atalar B^21^  2018  US  Retrospective | Craniospinal rad: 193 (94%)  Rad: post fossa 190 (92%)  The median (range) CSI dose was 36 (23.0–50.0) Gy; the median (range) boost dose to the posterior fossa was 18 (16.0–34.2) Gy, with a median (range) RT fraction size of 1.8 (1.5–2.0) Gy.  The total RT dose for the posterior fossa was a median (range) of 54 (35.2–72.0) Gy.  Chemo: 98 (48%) | Use of Chemo- Yes vs no, P=0.02 for DFS, P=0.03 for OS | . | CSI RT- Yes vs no, P<0.001 for DFS , P=<0.001 for OS  Time from surgery to RT days- ≤47 vs >47,P=0.04 for DFS, P=0.03 for OS  Total RT dose to post fossa <54 vs ≥54 GY- P<0.001 for DFS, P<0.001 for OS  Use of RT boost- Yes vs no, P<0.001 for DFS, p<0.001 for OS | craniospinal irradiation(CSI) yes vs no  DFS- RR 5.23 p<0.001,  OS- RR 5.32 p<0.001 | Surgical extent- Complete resection vs other, P=0.02 for DFS, P=0.01 for OS  Residual volume- ≤1.5 cm2 vs >1.5 cm2, P=0.04 for DFS P=0.02 for OS | . |
| Bloom H^36^  1990  UK  Retrospective | Rad: posterior fossa-45  55-58 Gy=15  41-52 Gy=30  Chemo+rad:20  Rad alone: 25 | Chemo + radiation therapy (vs radiation alone) (P<0.025) | . | Radiation dose to the posterior fossa of ≥55 Gy yes vs no. (P= 0.1) | . | Complete/subtotal resection (vs partial removal) (p<0.005) | . |
| Carrie C^22^  1994  France  Retrospective | Rad alone: craniospinal 79  Posterior fossa – 1  Chemo+rad: 75  median dose to the whole brain was 35 Gy (range, 0-45 Gy), that to the spinal axis was 35 Gy (range, 0-40 Gy), and that to the posterior fossa, 55 Gy (range, 42-60 Gy). | No chemo vs chemo-nonsig  Chemo-CCNU/VCR vs IF/CDDP/VCR, nonsig | . | Rad to posterior fossa-≥50 Gy vs <50, P= 0.09  Rad to whole brain-≥30 Gy vs <30, nonsig.  Radiation to spinal axis--≥30 Gy vs <30. P= 0.003 | spinal axis radiation dose>30Gy, RR 4.3 p=0.05  rad dose to posterior fossa, p=0.17 | Quality of resection-no residue vs residue-nonsig | Quality of resection no residue vs residue, p=0.13 |
| Carrie C^23^  1993 | Rad: whole brain-28  Cerebral fossa:29  Cerebral axis: 28  Chemo: 24  Chemo+rad:22 | Chemotherapy no vs yes, nonsig  Chemotherapy no vs 8/1 vs other chemo- nonsig | . | . | . | Resection quality complete vs incomplete, p<0.05 | . |
| Chan A^32^  2000  US  Retrospective | Rad: 32  Rad+chemo: 24  Rad dose craniospinal axis: median 36Gy  Rad dose post fossa=median 55Gy (52-60.4)  Median dose to the whole brain 36Gy (30.6-45) | chemotherapy-No vs yes, RR 2.86 CI (0.70-19.7), P=0.47 | . | Duration of radiotherapy-<48 vs ≥48days RR 1.86, CI(0.57-6.34), P=0.28 | . | Extent of surgery-complete vs less than complete, RR 10.9 CI(2.73-80.8), P=0.01 | Extent of surgery-complete vs less than complete, p= 0.02 |
| Chargari C^24^  2010  France  Retrospective | Rad: brain+spine-35 (97.1%)  Brain only: 1 (2.7%)  Chemo: 22 (59.5%)  Median whole brain dose 36Gy  Median spine dose 36Gy  Median boost dose 18 (16.2 – 19.8) | . | the use of chemotherapy-nonsig | . | . | . | . |
| Giordana M^25^  1995  Italy  Retrospective | Rad: 32  >50Gy to posterior fossa+whole CNS=14  30-40 Gy on post fossa=3  <30Gy or no rad therapy=15 | . | . | Radiotherapy>50Gy to posterior fossa+ whole CNS vs 30-40 Gy on post fossa vs <30Gy or no rad therapy (P=0.02) | . | Surgery-total vs subtotal- nonsig | . |
| Giordana M^35^  2005  Italy  Retrospective | 86 craniospinal neuraxis  (50 Gy to posterior fossa; 35 Gy to craniospinal neuraxis) | . | . | . | . | . | . |
| Hadi I^33^  2018  Germany  Retrospective | CSI of the entire brain and spine=21  Chemo+rad=20    median dose of 35.2Gy (range 23.4–39.0Gy  Boost to post fossa=single doses of 1.8 Gy to a median total dose of 19.8Gy (range 14.4–30.6Gy) | . | . | CSI technique-3D-RT vs 2D RT, P=0.272 for PFS, P=0.261 for OS  Duration of locoregional treatment (interval between tumor resection and end of irradiation, ≤ 73 days vs >73 days, P=0.049 for PFS, P=0.031 for OS | . | . | . |
| Herrlinger U^26^  2005  Germany  Retrospective | Craniospinal rad=16  Rad+chemo(adjuvant)=20  Median dose Rad only  posterior fossa 55 Gy (50–60 Gy)-(n = 16)  Whole brain 35 Gy (34–37.8 Gy)- (n = 15)  Spinal cord 35.5 Gy (34.6–37.8 Gy)-(n = 15)  Chemo+rad median dose  Posterior fossa 57.8 Gy (23.4–68 Gy) (n = 19)  Whole brain 35.2 Gy (23.2–37.8 Gy) (n = 19)  Spinal cord- 35.1 Gy (23.4–35 Gy) (n = 18) | Primary chemotherapy-yes vs no, RR 1.89 CI (0.95–4.86), P=0.068  Primary chemotherapy-yes vs no, RR 1.51 CI(0.92–2.69), P=0.10 | . | Time between surgery and radiotherapy- <5months vs ≥5 months, RR 0.86 CI(0.43–1.62), P=0.65  Time between surgery and radiotherapy- <5months vs ≥5 months, RR 1.07 CI (0.62–1.79), P=0.80 | . | Resection type-partial vs total, RR 1.01 CI (0.51–1.90), P=0.96 for OS  Resection type-partial vs total, RR 0.98 CI (0.57–1.62), P=0.95 for recurrence free survival | . |
| Kann B^27^  2017  US  Retrospective | 520 (69.2%) received chemo+rad, and 231 (30.8%) received RT alone.  477 patients with documented CSI doses  23 to <30 Gy=n=121  30 to 36 Gy=n=356  Other/Unknown Dose=n=274  Survival analysis  142 patients (30.3%) had RT, and 326 patients (69.7%) had CRT. | . | . | CRT vs RT, HR 0.42 CI (.27 to .64), P<0.001  CSI dose- 30 to 36 Gy vs (23 to <30 Gy), HR 1.20 CI (.72 to 1.99), P=0.49  Time-to-RT, weeks (continuous), HR 0.97, CI (.92 to 1.02), P=0.29 | CRT vs RT, HR 0.53 CI(.32 to .88), P=0.01  CSI dose- 30 to 36 Gy vs (23 to <30 Gy), HR 0.91 CI(.51 to 1.64), P=0.76  Time-to-RT, weeks (continuous), HR 0.97 CI(.91 to 1.02), P=0.23 | Extent of resection-macroscopic residual tumor vs no macroscopic residual tumor, HR 0.41 CI(.05 to 3.31), P=0.40 | Extent of resection-macroscopic residual tumor vs no macroscopic residual tumor, HR 0.34 CI(.04 to 2.80), P=0.32 |
| Kunschner L^28^  2001  US  Retrospective | Craniospinal rad=26  Whole brain rad=2  Post fossa boost=46  Adjuvant chemo=6 | use of adjuvant chemotherapy-nonsig | . | . | . | extent of resection GTR vs others-nonsig | . |
| Lai R^29^  2008  US  Retrospective | No radiation=67 (14.76)  Rad given=377 (83.04)  Unknown=10 (2.20) | . | . | Radiation  No radiation-reference  Radiation given, HR 0.52 CI(0.34–0.80), P=0.003 | Radiation  No radiation-reference  Radiation given, HR 0.52 CI (0.33–0.82),P=0.005 | Surgical resection  Biopsy-reference,  Subtotal resection, HR 0.48 CI (0.20–1.16), P=0.10  Gross total resection, HR 0.35 CI(0.15–0.82), P=0.012  Surgery NOS, HR 0.56 CI(0.24–1.29), P=0.17 | Surgical resection  Biopsy-reference  Subtotal resection, HR 0.49 CI(0.20–1.20), P=0.12  Gross total resection, HR 0.33 CI (0.14–0.78), P=0.012  Surgery NOS, HR 0.14 CI(0.04–0.52),P=0.003 |
| Le Q T^30^  1997  US  Retrospective | Craniospinal irradiation=34  Most patients treated after 1979 also received chemo.  Chemo=23  Post fossa dose  Conventional 54-62,4Gy (median 55.8Gy)  Hyperfractionated: 70-72 (median 72Gy) | . | . | Radiation dose to the post fossa-P=0.07 | Posterior fossa dose, low vs high, p>0.10 | Extent of surgical resection- P=0.06 | GTR, P>0.10 |
| Padovani L^34^  2007  France  Retrospective | Radiotherapy=246  Radiochemotherapy= 142  Chemotherapy= 252  Radiotherapy + chemo=253  Brain rad dose ≥30Gy 249  Spinal rad dose ≥30Gy=249  Posterior fossa radiation dose ≥50 Gy=250  Median doses delivered were 35 Gy to the brain, 35 Gy to the spinal area, and 54 Gy to the PCF. | Chemotherapy no vs yes, p=0.95 | . | Brain radiation dose (Gy) >29 vs ≤29, p=0.48  Spinal radiation dose (Gy), ) >29 vs ≤29, P=0.0054  Posterior fossa radiation dose (Gy) ≥50 vs <50 P<0.0001  Radiation duration (d) >45 vs ≤45 p=0.756 | Dose of rad to the posterior fossa <50Gy RR 2.7 CI(1.3–5.8), P=0.009 | Surgical residue, no vs yes, P=0.08  Date of surgery after 1990 vs before 1990, P=0.85 | . |
| Rodriguez FJ^31^  2007  US  Retrospective | Post-operative radiation (85%) and chemotherapy (27%).  Sixty-five of the 74 patients received adjuvant radiotherapy, chemotherapy, or a combination of the 2. Specifically, 45 patients received radiotherapy to the craniospinal axis, 2 patients received chemotherapy only, and 18 patients received both.  Adjuvant chemo=20 | Adjuvant chemotherapy-nonsig | . | . | . | . | . |

**Abbreviations in this table:**

RT: radiotherapy, CRT: chemoradiation, CSI: craniospinal irradiation, NOS: not otherwise specified, GTR: gross total resection, OS: overall survival, RFS: recurrence free survival, PFS: progression free survival, DFS: disease free survival, EFS: event free survival, Nonsig- non-significant, HR: hazard ratio, RR: risk ratio, CI: confidence interval, CCNU/VCR vs IF/CDDP/VCR: CCNU: chloroetyl cyclohexyl~nitrosourea, VCR: vincnstine, CDDP: cisplatinurn; IF ifosfamide
