# Supplementary material for: Prognostic factors related to overall survival in adolescent and young adults with medulloblastoma: A systematic review
Source: Neurooncol Adv. 2022 Feb 9;4(1):vdac016. doi: 10.1093/noajnl/vdac016 (PMC9161716; doi:10.1093/noajnl/vdac016)
Supplement: vdac016_suppl_Supplementary_Appendix [file vdac016_suppl_supplementary_appendix.docx]

**Appendix 1: Sample of search strategy**

**Ovid MEDLINE(R) and Epub Ahead of Print, In-Process & Other Non-Indexed Citations and Daily**1946 to August 17, 2020
Search Strategy:

| **#** | **Searches** | **Results** |
| --- | --- | --- |
| 1 | Medulloblastoma/ | 6897 |
| 2 | (medulloblastoma? or medullomyoblastoma? or medullomyoblastomas or blastoma medullae or medullo blastoma).tw,kf. | 8411 |
| 3 | or/1-2 | 9997 |
| 4 | Adolescent/ | 2028675 |
| 5 | Adult/ | 5004246 |
| 6 | (adolescen* or teen* or preteen* or pre-teen* or youth? or young person? or young people or adult?).mp. | 6520365 |
| 7 | (AYA or AYAs).tw,kf. | 1532 |
| 8 | or/4-7 | 6520413 |
| 9 | prognosis.sh. or diagnosed.tw. or cohort:.mp. or predictor:.tw. or death.tw. or exp models, statistical/ | 2745015 |
| 10 | prognos*.tw,kf. | 619020 |
| 11 | or/9-10 | 3006570 |
| 12 | Survival/ | 4725 |
| 13 | Cancer Survivors/ | 3787 |
| 14 | Survival Rates/ | 173268 |
| 15 | progression-free survival/ | 3226 |
| 16 | disease-free survival/ | 74305 |
| 17 | surviv*.tw,kf. | 1154096 |
| 18 | or/12-17 | 1227493 |
| 19 | 3 and 8 and 11 and 18 | 771 |
| 20 | Epidemiologic studies/ | 8382 |
| 21 | exp case control studies/ | 1096482 |
| 22 | exp cohort studies/ | 2020140 |
| 23 | Case control.tw. | 126484 |
| 24 | (cohort adj (study or studies)).tw. | 209981 |
| 25 | Cohort analy$.tw. | 8185 |
| 26 | (Follow up adj (study or studies)).tw. | 49506 |
| 27 | (observational adj (study or studies)).tw. | 108791 |
| 28 | Longitudinal.tw. | 247649 |
| 29 | Retrospective.tw. | 538667 |
| 30 | Cross sectional.tw. | 358439 |
| 31 | Cross-sectional studies/ | 334381 |
| 32 | or/20-31 | 3039162 |
| 33 | randomized controlled trial.pt. | 511241 |
| 34 | clinical trial.pt. | 524284 |
| 35 | randomi?ed.ti,ab. | 631465 |
| 36 | placebo.ti,ab. | 216030 |
| 37 | dt.fs. | 2227042 |
| 38 | randomly.ti,ab. | 340123 |
| 39 | trial.ti,ab. | 603058 |
| 40 | groups.ti,ab. | 2108445 |
| 41 | or/33-40 | 4959475 |
| 42 | animals/ | 6650952 |
| 43 | humans/ | 18643860 |
| 44 | 42 not (42 and 43) | 4692606 |
| 45 | 41 not 44 | 4324596 |
| 46 | 32 or 45 | 6392134 |
| 47 | 3 and 8 and 11 and 18 and 46 | 515 |
| 48 | limit 47 to english language | 476 |
